# Large cryptic genomic rearrangements with apparently normal karyotypes detected by array-CGH

**DOI:** 10.1186/s13039-014-0082-7

**Published:** 2014-11-19

**Authors:** Eleonora Di Gregorio, Elisa Savin, Elisa Biamino, Elga Fabia Belligni, Valeria Giorgia Naretto, Gaetana D’Alessandro, Giorgia Gai, Franco Fiocchi, Alessandro Calcia, Cecilia Mancini, Elisa Giorgio, Simona Cavalieri, Flavia Talarico, Patrizia Pappi, Marina Gandione, Monica Grosso, Valentina Asnaghi, Gabriella Restagno, Giorgia Mandrile, Giovanni Botta, Margherita Cirillo Silengo, Enrico Grosso, Giovanni Battista Ferrero, Alfredo Brusco

**Affiliations:** Department of Medical Sciences, University of Torino, via Santena 19, 10126 Torino, Italy; Città della Salute e della Scienza University Hospital, Medical Genetics Unit, Turin, Italy; Department of Public Health and Pediatrics, University of Torino, Turin, Italy; Department of Neuropsychiatry, University of Torino, Turin, Italy; Laboratory of Molecular Genetics, Città della Salute e della Scienza University Hospital, Turin, Italy; Medical Genetics unit, San Luigi University Hospital, Orbassano, Italy; Department Clinical and Biological Sciences, University of Torino, Torino, Italy; Department of Pathology, Città della Salute e della Scienza University Hospital, Turin, Italy

**Keywords:** GTG-banding, Array-CGH, Unbalanced derivative chromosomes, CNV, Genomic rearrangement, Intellectual disability

## Abstract

**Background:**

Conventional karyotyping (550 bands resolution) is able to identify chromosomal aberrations >5-10 Mb, which represent a known cause of intellectual disability/developmental delay (ID/DD) and/or multiple congenital anomalies (MCA). Array-Comparative Genomic Hybridization (array-CGH) has increased the diagnostic yield of 15-20%.

**Results:**

In a cohort of 700 ID/DD cases with or without MCA, including 15 prenatal diagnoses, we identified a subgroup of seven patients with a normal karyotype and a large complex rearrangement detected by array-CGH (at least 6, and up to 18 Mb). FISH analysis could be performed on six cases and showed that rearrangements were translocation derivatives, indistinguishable from a normal karyotype as they involved a similar band pattern and size. Five were inherited from a parent with a balanced translocation, whereas two were apparently *de novo*. Genes spanning the rearrangements could be associated with some phenotypic features in three cases (case 3: *DOCK8*; case 4: *GATA3, AKR1C4*; case 6: *AS/PWS* deletion, *CHRNA7*), and in two, likely disease genes were present (case 5: *NR2F2, TP63, IGF1R*; case 7: *CDON*). Three of our cases were prenatal diagnoses with an apparently normal karyotype.

**Conclusions:**

Large complex rearrangements of up to 18 Mb, involving chromosomal regions with similar size and band appearance may be overlooked by conventional karyotyping. Array-CGH allows a precise chromosomal diagnosis and recurrence risk definition, further confirming this analysis as a first tier approach to clarify molecular bases of ID/DD and/or MCA. In prenatal tests, array-CGH is confirmed as an important tool to avoid false negative results due to karyotype intrinsic limit of detection.

## Background

GTG-banding karyotype is a standard procedure in the diagnosis of patients with unexplained intellectual disability/developmental delay (ID/DD), autism spectrum disorders (ASD), and multiple congenital anomalies (MCA) [1]. The limit for the detection of genomic rearrangements is estimated above 5–10 Mb at the 500–550 band level, at least in regions where the band pattern is distinctive [2]. Karyotyping is also widely used in prenatal testing even if its resolution is lower due to a more compact chromatin structure.

The detection of submicroscopic rearrangements by array-CGH has increased the diagnostic yield of patients with ID/DD and/or MCA of 15-20% [3–5], due to array-CGH higher resolution vs. karyotyping (50–100 kb on a 60 K Agilent platform) [3,6]. Indeed, since its introduction, array-CGH analysis has evidenced that karyotyping can also miss large (>7 Mb) and very large (>10 Mb) rearrangements [2]. An estimate of the number of these overlooked rearrangements is still unknown. These are likely to be mainly derivative chromosomes with deletions and duplications, involving chromosomal regions with a similar banding pattern and size. These anomalies can result from parental balanced translocations that malsegregate at meiosis. Given that ~0.5% of the general population is estimated to be carrier of a balanced rearrangement [7], derivative chromosomes apparently normal at karyotype may be more common than expected. Whereas balanced translocation are associated with infertility and recurrent miscarriages [8], the clinical consequences of derivative chromosomes can be lethal or lead to complex severe phenotypes.

This study includes seven patients presenting with complex developmental anomalies, an apparently normal karyotype and an unexpected large (>6 Mb) chromosomal rearrangement detected by array-CGH.

## Results

**Figure 1:**
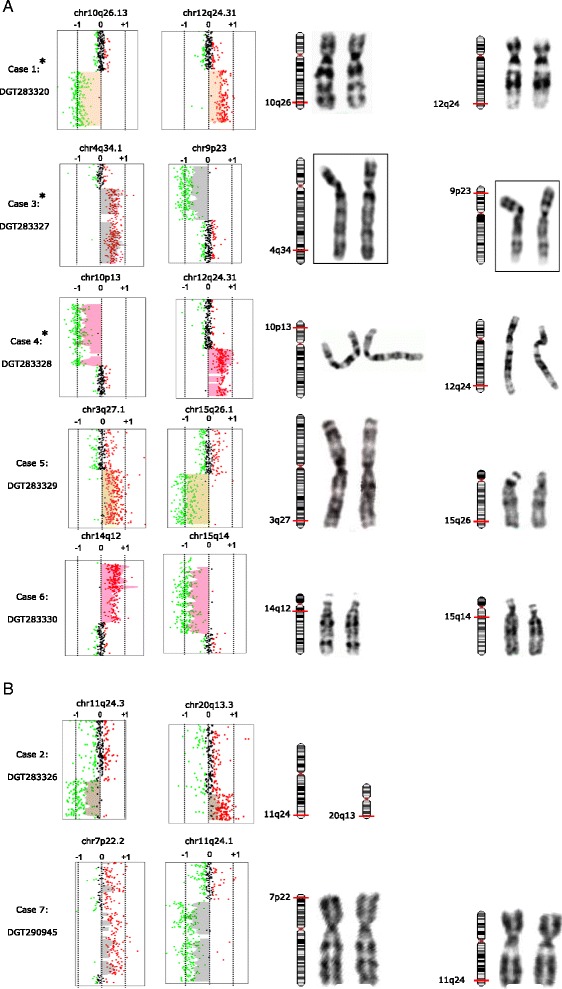
**Array-CGH and karyotyping in cases with inherited and** ***de novo*** **chromosomal rearrangements. Panel A.** Array-CGH analysis resulting in telomeric rearrangements on two different chromosomes (left). On the X-axis, the log ratio is reported (log_2_ intensity of [Cy5 fluorochrome/Cy3 fluorochrome)]. Expected values are from −0.7 to −1 for a deletion (green dots), 0 for normal (black dots), and +0.5 to +1 for a duplication (red dots). On the right side, GTG-banding of the chromosomes involved in the structural rearrangements. In cases marked by an asterisk, the chromosomes on the right are from the parent carrying the cryptic translocation, because patient karyotype image was not available. Breakpoints are indicated by red bars on chromosomal ideograms. In **panel B**, array-CGH analysis displays telomeric rearrangements in probands with *de novo* rearrangements.

**Figure 2:**
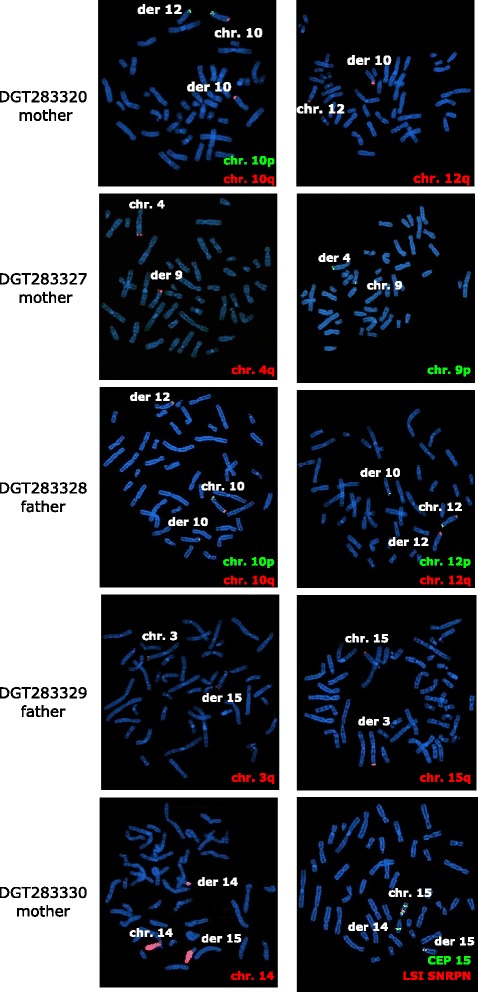
**FISH analysis of the parents carrying balanced translocation.** FISH probes hybridize on the normal homologues as well as on derivative chromosomes. Probes used in these analysis were the following: 3q SpectrumOrange (05 J04-003, Vysis TelVysion probe, Abbot, Illinois, USA), 4q SpectrumOrange (05 J04-004, Vysis TelVysion probe), 10p SpectrumGreen (05 J03-010, Vysis TelVysion probe), 10q SpectrumOrange (05 J04-010, Vysis TelVysion probe), 12p SpectrumGreen (05 J03-012, Vysis TelVysion probes), 12q SpectrumOrange (05 J04-012, Vysis TelVysion probe),15q SpectrumOrange (05 J04-015, Vysis TelVysion probe), painting 14 (LPP14R, CYTOCELL, Cambridge, UK), Prader-Willi/Angelman Region probe -LSI SNRP Spectrum orange/CEP15 (D15Z1) SpectrumAqua/LSI PML SpectrumGreen (05 J26-027, Vysis, Abbot).

In our survey of 700 patients with ID/DD and/or MCA analyzed by array-CGH as pre or postnatal test from 2008 to 2013, we identified 156 pathogenic or likely pathogenic rearrangements (manuscript in preparation). Seven cases from this cohort showed at least two large subtelomeric rearrangements - a deletion and a duplication spanning from 4.4 to 18 Mb - compatible with a derivative chromosome (Figures 1 and 2, and Table 1). In each case, one of the two genomic rearrangement was at least 6 Mb, although karyotype was reported normal. We confirmed the presence of a derivative chromosome by FISH in six subjects, and demonstrated it was inherited from a parent with a balanced translocation in five cases (Figure 2). In the remaining two, the rearrangement was apparently *de novo* (Table 1). The band pattern and sizing of the exchanged genomic region was very similar in all analyzed cases (Figure 1). The balanced chromosomal anomaly in the parent was also undetectable by karyotyping in four out of the five transmitted cases (Cases 1–5, Figure 1A). Rearrangements were always associated with complex developmental defects, summarized in Table 2 and described below.

**Table 1:** **Summary of the seven patients with large rearrangement missed by karyotype**

**Patient code (Decipher)**		**Gender**	**Array-CGH (GRCh37/hg19)**	**Minimal region (Mb)**	***de novo*** **/inherited**	**Cell source for karyotype**
**1**	DGT283320	M	arr 10q26.13q26.3(124,500,982-135,404,471)x1, 12q24.31q24.33(125,178,836-133,819,092)x3	10.9: 8.6	maternal translocation	Amniocytes
**2**	DGT283326	F	arr 11q24.3q25(128,728,456-134,868,407)x1, 20q13.3(58,442,781-62,893,189)x3,	6: 4.4	*de novo*	Periph. Blood
**3**	DGT283327	M	arr 4q34.1q35.2(172,930,618-190,896,674)x3, 9p24.3p23(271,257-12,907,826)x1, 14q21.1(43,881,311-44,623,069)x3	18: 12.6: 0.74	maternal translocation	Periph. Blood
**4**	DGT283328	M	arr 10p15.3p13(148,206-12,211,671)x1, 12q24.31q24.33(121,572,578-133,767,986)x3	12: 12.2	paternal translocation	Periph. Blood
**5**	DGT283329	M	arr 3q27.1q29(184,428,168-197,840,339)x3, 15q26.1q26.3( 90,857,664-102,383,473)x1	13: 11.5	paternal translocation	Amniocytes
**6**	DGT283330	M	arr 2p12p11.2(82,510,808-84,804,525)x1, 14q11.2q12(20,472,548-31,139,579)x3, 15q11.1q14(20,102,541-35,758,169)x1	2.2: 10.7: 15.6	maternal translocation	Periph. Blood
**7**	DGT290945	F	arr 7p22.3p22.2 (92.532-4.176.031)x3, 7p22.3p22.2 (7.044.310-15.709.683)x3, 11q24.1q25(122.467.330-134.868.407)x1	4: 8.6: 12.4	*de novo*	Chorionic villi

**Table 2:** **Genes involved in the rearrangements and their associated phenotypes**

**Case**	**Age**	**Structural anomaly**	**Genes involved**	**OMIM associated genes** ^**a**^	**Likely pathogenic genes (haploinsufficiency)**	**Patient’s features corresponding to the pathogenic genes**
**1**		del 10q26.13q26.3	76	3 (AR)	-	
	4 yrs	dup 12q24.31q24.33	54	1 (ADdn)	-	
				3 (AR)		
**2**	10 yrs	del 11q24.3q25	29	1 (ADdn)	-	
				4 (AR)		
		dup 20q13.3	90	7 (ADh)	-	
**3**	6 yrs	del 9p24.3p23	29	1 (ADdn)	*SLC1A1* (susceptibility to schizophrenia and psychotic disorder)	Patient too young to verify symptoms
				4 (AR)	*SMARCA2* (Nicolaides-Baraitser synd.; dominant negative)	Intellectual disability, delayed speech, psychomotor development stooped posture and seizures
				1 (AD)	*DOCK8* (Intellectual disability)	Intellectual disability
		dup 4q34.1q35.2	53	9 (AR)	*CCDC111* (susceptibility to high myopia)	Patient too young to verify symptoms
		dup 14q21.1	Gene desert	-		
**4**	15 yrs	del 10p15.3p13	73	3 (ADh)	*GATA3* (hypoparathyroidism, sensorineural deafness, and renal insufficiency)	Congenital hypoparathyroidism, deafness and renal disease
				1 (AR)	*AKR1C4* (46XY sex reversal)	Cryptorchidism, hypospadia
					*DHTKD1* (Charcot-Marie-Tooth type 2Q)	Patient too young to verify symptoms
		dup 12q24.31q24.33	108	1 (ADh)	*P2RX2* (hearing loss)	May duplication affect hearing ability?
				1 (ADdn)		
				9 (AR)		
**5**	TOP	del 15q26.1q26.3	30	4 (ADh)	*CHD2* (Childhood onset encephalopathy)	-
					*NR2F2* (Heart defects)	Ventricular septal defect
				7 (AR)	*IGF1R* (Growth retardation)	IntraUterine Growth Retardation Heart malformation?
		dup 3q27.1q29	114	4 (ADh)	*MEF2A* (Coronary artery disease)	May duplication cause limb anomalies?
				1 (AD?)	*TP63* (Heterodactily, ectodermal dysplasia, cleft lip palate syndrome 3)	
				9 (AR)		
**6**	1 yr	del 2p12p11.2	1	1 (AR)		
		del 15q11.1q14	60	5 (AR)	*NPIA1* (Spastic paraplegia)	Patient too young to verify symptoms
					*MKRN3* (Precocious puberty)	Patient too young to verify symptoms
					*MAGEL2* (Prader-Willy like, imprinted)	Maternally inherited deletion
				7 (ADh)	*NDN, SNRP, UBE3A* (Angelman/Prader-Willy, imprinted)	Angelman features (maternally inherited deletion)
					*CHRNA7* (15q13.3 syndrome)	Myoclonic seizures
		dup 14q11.2q12	60	4 ADh	*ANG* (Amyotrophic lateral sclerosis)	Patient too young to verify symptoms
				1 (ADgain)	*CHD8* (Autism)	Duplication may be associated with psychomotor developmental delay
				2 (AD?)	*MYO6* (Atrial septal defect)	Pervious foramen ovale
				6 (AR)	*FOXG1* (Rett-like syndrome)	Duplication may be associated with neurocognitive impairment
		del 11q24.1q25	58	3 (ADh)	*SCN3B* (Brugada syndrome)	-
**7**	TOP			1 (AD?)	*CDON* (Holoprosencephaly type 11)	Brain malformation
				10 (AR)	*KIRREL3* (Intellectual disability)	-
		dup 7p22.3p22.2	42	4 (AR)	*-*	

Notes: ^a^AR: autosomal recessive; ADdn: autosomal dominant, dominant negative effect; ADh: autosomal dominant, haploinsufficiency; AD?: autosomal dominant, unknown effect; ADgain: autosomal dominant with gain of function. TOP: Termination of Pregnancy.

### Case 1. DGT283320

**Figure 3:**
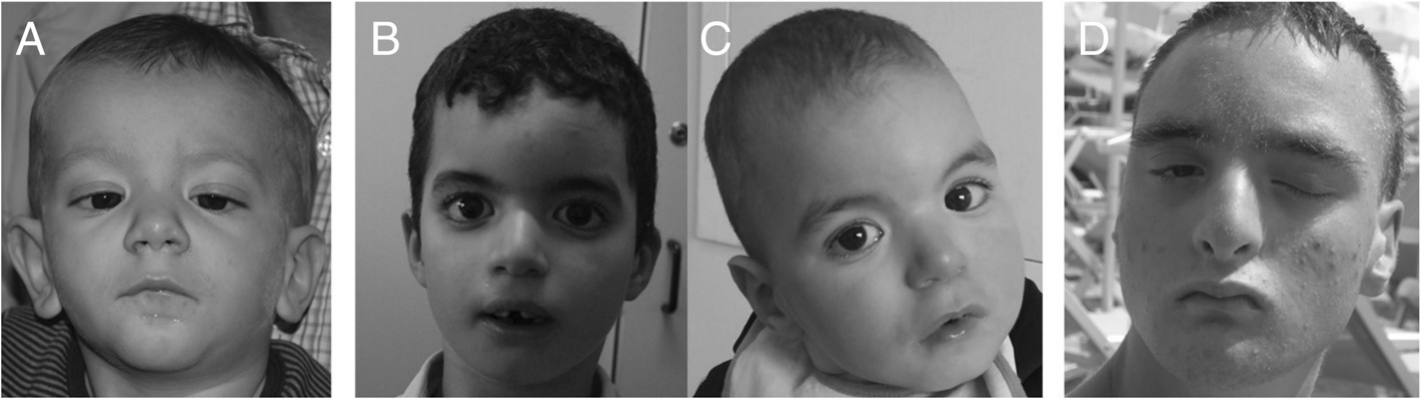
**Dysmorphological features of cases 1, 3 and 4. A.** Subject 1 at 18 months, shows mild facial dysmorphisms (triangular face, epicanthus, thin upper lip). **B.** Subject 3 (proband) at 6 yrs., dolico-trigonocephaly, thick eyebrows, mild synophris, mid-face hypoplasia. **C.** Younger brother of subject 3 at 2 yrs. showing similar dysmorphisms. **D.** Subject 4 at 14 yrs., showing bristly hair, left palpebral ptosis, broad nasal root, small ears.

The proband was a 3-year-old boy, fifth child of non-consanguineous parents. He was born by Cesarean section at 39 weeks of gestation after an uneventful pregnancy. Neonatal weight was 2,740 g (3^rd^ percentile [9]), length 47 cm (3^rd^ percentile), and Occipital Frontal Circumference (OFC) 34.5 cm (50^th^ percentile). Following birth, he needed immediate ventilation support (APGAR: 5/9). Since birth, he has shown severe hypotonia and feeding difficulties. Extensive metabolic workup was normal (levels of plasmatic and urinary amino acids, urinary organic acids profile, beta-N-acetyl-glucosamine, cerebroside beta-galactoside, arylsulfatase and chitotriosidade activity, lactic dehydrogenases, creatine kinase (CK) and serum creatinine). Isoelectrophoretic analysis ruled out main congenital disorders of glycosylation. Cerebral MRI detected hypoplastic corpus callosum, without other malformations. Neurological examination at 9 months revealed severe axial hypotonia, poor eye-hand coordination, and exotropia; he showed only mild facial dysmorphisms, namely a triangular face and thin upper lip (Figure 3A).

The prenatal karyotype was normal (46,XY). Postnatal array-CGH analysis on lymphocytes detected a 10.9 Mb deletion at 10q26.13q26.3 and an 8.6 Mb duplication at 12q24.31q24.33 (Figure 1A, Tables 1 and 2). FISH analysis with probes specific for the subtelomeric regions of the long arms of chromosome 10 and 12 revealed a reciprocal balanced translocation in the mother (Figure 2). The balanced rearrangement in the mother was not evident upon GTG-banding analysis (Figure 1A, right).

### Case 2. DGT283326

Female born from non-consanguineous healthy parents, after an uneventful pregnancy. Psychomotor delay and failure to thrive were reported. Delayed speech development was observed (first words at 4 yrs). At 7 yrs, clinical examination showed stature and weight below the 3^rd^ centile, facial dysmorphisms (long, deeply grooved philtrum, thin upper lips), supernumerary nipples, pectus excavatum, hypertrichosis. The patient showed moderate intellectual delay.

Patient postnatal karyotyping, performed on blood, was reported normal (46,XX) (GTG-banding images were not available). Array-CGH analysis on the DNA extracted from proband whole blood showed a 6 Mb deletion at chromosome 11q24.3q25, and a 4.4 Mb duplication at 20q13.3 (Figure 1B, Tables 1 and 2). FISH analysis on the parents, using probes for the subtelomeric regions of the long arms of chromosome 11 and 20 was normal, suggesting a *de novo* event.

### Case 3. DGT283327

The parents of this male proband were first cousins. He was, initially referred for DD at the age of 18 months. Family history was negative. He was born at full-term by Caesarean section after an uneventful pregnancy, with normal auxometric parameters. After birth, he received oxygen supplementation for mild respiratory distress (APGAR score 7/9). Cardiac ultrasound disclosed a modest ventricular septal defect that spontaneously resolved in a few months. Bilateral cryptorchidism and a metopic ridge with OFC at the 25^th^ centile were also reported. Brain MRI detected a corpus callosum hypoplasia and several high-intensity lesions in the bilateral periventricular white matter T2-weighted imaging, due to perinatal hypoxia.

Methylation test for Prader-Willi syndrome and metabolic workup was normal. The patient was re-evaluated at 5 yrs during hospitalization for generalized seizures that occurred with fever. ElectroEncephaloGraphy (EEG) showed multifocal epileptic activities, and the patient was under valproic-acid therapy. He had a normal OFC (50 cm- 25^th^ centile), dolico-trigonocephaly, thick eyebrows, mild synophrys, mid-face hypoplasia, retrognathia, and flat feet (Figure 3B). He started walking independently at the age of 4.5 yrs, but never developed any language skill. His younger brother presenting a similar phenotype (developmental delay, dolico-trigonocephaly, synophris, Figure 3C).

Patient postnatal karyotyping, performed on blood was reported as normal (46,XY) (GTG-banding images were not available). The subsequent array-CGH analysis on the DNA extracted from proband whole blood showed three rearrangements: an 18 Mb duplication at 4q34.1q35.2, a 12.6 Mb deletion at 9p24.3p23 and a 742 kb duplication at 14q21.1 (Figure 1A, Tables 1 and 2). The latter encompassed a gene desert region.

FISH analysis on the parents, using probes for the subtelomeric regions of the long arms of chromosome 4 and 9 revealed a reciprocal balanced translocation in the mother (Figure 2). The balanced rearrangement in the mother was not evident upon GTG-banding analysis (Figure 1A, right).

### Case 4. DGT283328

The patient was the second son of unrelated parents. Family history was negative for ID/DD and/or MCA, and the pregnancy was uneventful. A low weight, left palpebral ptosis, hypospadias, and bilateral cryptorchidism were noted at birth. Further investigations disclosed right chorioretinal coloboma, bilateral mixed hearing loss, one sacral hemivertebra and bilateral bladder-ureteric reflux associated with right kidney dysplasia. The patient presented intermittent hypocalcaemia due to congenital hyperparathyroidism. A tethered spinal cord was diagnosed and surgically corrected at the age of 4 yrs. The patient was referred for genetic evaluation at the age of 13 yrs, during hospitalization for seizures. Brain MRI detected left hippocampus hypoplasia. He displayed profound intellectual disability (he was not able to walk unsupported and language was absent) and microcephaly; moreover he showed striking dysmorphisms, including bristly hair, left palpebral ptosis, broad nasal root, and a small ears (Figure 3D).

Patient postnatal karyotyping, performed on blood, was reported as normal (46,XY) (GTG-banding images were not available). The subsequent array-CGH analysis on the DNA extracted from proband whole blood showed a 12 Mb deletion at 10p15.3p13 and a 12.2 Mb duplication at 12q24.31q24.33 (Figure 1A, Tables 1 and 2). FISH analysis on the parents confirmed the presence of a derivative chromosome between chromosomes 10 and 12, with a balanced translocation inherited from the father (Figure 2). The balanced rearrangement in the father was not evident at GTG-banding analysis (Figure 1A, right).

### Case 5. DGT283329

A pregnant woman was referred for very low levels of maternal serum PAPP-A, and multiple miscarriages. Pregnancy was interrupted at 21 weeks of gestation for severe Intrauterine Growth Restriction (IUGR) (<5^th^ centile) and multiple developmental defects. Postmortem pathologic evaluation confirmed IUGR, and revealed midline fused eyebrows, marked hypognathia, nucal edema, short limbs, thymus hypoplasia, ventricular septal defect, and pulmonary and cerebellar hypoplasia. A diagnosis of Cornelia de Lange syndrome was suggested.

Prenatal karyotyping on amniocytes (46,XY) (Figure 1A, right), molecular analysis of *NIPBL* and *SMC1*, hypomethylation H19 and uniparental disomy of chromosome 7 were normal.

Array-CGH analysis performed on DNA extracted from autoptical fetal tissue showed a 13 Mb duplication at 3q27.1q29 and a 11.5 Mb deletion at 15q26.1q26.3 (Figure 1A, Tables 1 and 2). FISH analysis on the parents revealed a balanced translocation inherited from the father (Figure 2).

### Case 6. DGT283330

First male child of non-consanguineous parents. Twin pregnancy with intrauterine death of one fetus at 6 weeks of gestation. Prenatal first trimester biochemical screening was normal. He was born by Caesarean section at 37 weeks of gestation. At birth, weight was 2,180 g (10^th^ percentile), length 42.2 cm (<3^rd^ percentile), OFC 34 cm (75^th^ percentile), and APGAR score 4/8. He showed hypotelorism, bulbous nose, long and smooth philtrum and mild micrognathia. At 7 days, the patient presented with seizures. Right multifocal spike and wave anomalies, along with left occipital-temporal anomalies were detected by EEG, requiring multi-pharmacological treatment. Cerebral ultrasound and MRI revealed hypoplastic corpus callosum. Renal ultrasound showed bilateral pyelectasis and hydronephrosis, and echocardiogram revealed a patent foramen ovale. Cortical auditory evoked potentials and visually evoked potentials gave normal results. Neurological follow-up at 11 months of age revealed DD. The child experienced recurrent myoclonic seizures during the first year of life, and needed multidrug antiepileptic treatment to clinically control seizures. Despite the pharmacological therapy, multifocal spike and wave anomalies persisted at follow-up EEG.

Patient postnatal karyotyping was normal (46,XY) (Figure 1A, right). Array-CGH analysis showed three rearrangements: a 2.2 Mb deletion at 2p12p11.2, a 10.7 duplication at 14q11.2q12 and a 15.6 Mb deletion at 15q26.1q26.3 (Figure 1A, Tables 1 and 2). FISH analysis on the parents revealed a balanced translocation in the mother (Figure 2).

### Case 7. DGT290945

A woman underwent pregnancy termination after ultrasonography diagnosis of fetal developmental defects. Fetal autopsy revealed a female fetus with IUGR, nuchal and occipital edema, flat nose, low-set ears, hypertelorism, thymus hypoplasia, preductal aortic arch and left heart hypoplasia, lung hypoplasia, corpus callosum agenesis, thoracic hemivertebrae, and short limbs.

Prenatal karyotyping on chorionic villi was normal (46,XX) (Figure 1B, right). Array-CGH analysis performed on DNA extracted from autoptic tissue from the fetus showed a ~13 Mb duplication at 7p22.3p22.2 and a 12.4 Mb deletion at 11q24.1q25 (Figure 1B, Tables 1 and 2). FISH analysis revealed the translocation was apparently *de novo*.

## Discussion

In our cohort of ID/DD and/or MCA cases, routinely analyzed by array-CGH in pre or postnatal tests, we identified seven cases with derivative chromosomes missed by karyotyping, even if involving at least one region above 6 Mb. Large rearrangements detected by array-CGH missed by karyotyping have been previously reported [2], but our data suggest they are more common than expected, accounting for ∼ 4.5% of pathogenic array-CGH anomalies in our cohort (7/156). Moreover, three of our cases were prenatal diagnoses, out of a total of 15. Although these numbers are limited, it is important to note the utility of array-CGH in the presence of ultrasound anomalies, even with an apparently normal karyotype [10].

In five of the seven cases, we detected a balanced translocation in an healthy parent. In two, the derivative chromosome was apparently *de novo*, suggesting a germinal mosaic translocation in one parent. The translocation was also not detectable upon karyotyping in four out of the five cases, and had to be confirmed using telomeric FISH. Comparison of the two translocated chromosomes showed that the band pattern and sizing were highly similar. The rearrangements were therefore not detected using standard karyotyping due to the intrinsic technical limits of this analysis.

All cases had complex congenital anomalies and, in most, disease associated genes or disease candidate genes could be tracked in the deleted/duplicated segment. However, even if these rearrangements were large, the number of causative genes was always limited from one to four; in two patients no disease gene was presently annotated in the deletion/duplication. This suggests a minority of genes in our rearrangements were dose sensitive, and/or the pathogenicity of deleted/duplicated genes remains to be discovered.

Neurodevelopmental anomalies in patient 3 could be associated with the deletion encompassing *DOCK8* (autosomal dominant mental retardation); *SMARCA2,* causing the autosomal dominant Nicolaides-Baraitser syndrome, may also have a role in the pathology, although reported mutations in this gene act as dominant negative [11,12]. In patient 4, the phenotype could be explained by the deletion of two genes: *GATA3* (hypoparathyroidism, sensorineural deafness, and renal insufficiency) and *AKR1C4* (46,XY sex reversal) [13,14]. Patient 6 was an Angelman syndrome phenocopy with atypical seizures. Indeed, he carried a large deletion spanning the AS/PWS critical region (maternally inherited) and the *CHRNA7* gene related to 15q13.3 deletion syndrome, associated with intellectual disability and epilepsy. He also carried a 10.7 Mb duplication with possible involvement in the phenotype of the duplicated *CHD8,* implicated in autism [15], and *FOXG1* (infantile growth retardation and epilepsy) [16–19].

In two additional patients, genes within the rearrangement were suggestive: in case 5, a duplication of *TP63* may explain limb anomalies. Indeed, *TP63* mutations cause at least five different types of ectodermal dysplasia syndromes, a non-syndromic split-hand/foot malformation (SHFM4) and non-syndromic cleft lip [20–23]; however its duplication has never been reported in a particular pathology. In the same patient, ventricular septal defects may be due to the *NR2F2* deletion, a gene recently associated with non-syndromic atrioventricular septal defects (AVSDs) [24]. Finally, growth retardation may be caused by *IGF1R* haploinsufficiency. This gene has an important role in fetal growth and skeletal development, and Insulin-like Growth Factors (IGFs) have also been demonstrated to be involved in limb morphogenesis [25].

In case 7, brain malformations may be associated with *CDON* deletion, which is mutated in holoprosencephaly type 11 [26].

## Conclusions

Large rearrangements above 6 Mb may remain undetected by karyotyping analysis, but can be an important cause of ID/DD and/or MCA, and be troublesome events in prenatal tests.

## Methods

### Patients

From 2008 to 2013, we assembled a cohort of over 700 ID/DD and/or MCA patients from the Pediatric Genetics Unit and the Medical Genetics Unit of the “Città della Salute e della Scienza” University Hospital (Torino, Italy). Fifteen cases were prenatal diagnoses. Seven patients presented a large rearrangement between 4.4 to 18 Mb, detected by array-CGH, but not revealed by karyotyping.

### Karyotyping and array-CGH

Karyotyping on GTG-banded chromosomes from patients was performed on chorionic villi (1 patient), amniocytes (2 patients), and cultured lymphocytes (4 patients) according to standard protocols. Array-CGH was performed using a 60 K whole-genome oligonucleotide microarray (2.1 kb average probe spacing; 1.8 kb in Refseq genes) following the manufacturer’s protocol (Agilent Technologies, Santa Clara, California, USA). Slides were scanned using a G2565BA scanner, and analyzed using Agilent CGH Analytics software ver. 4.0.81 (Agilent Technologies Inc.) with the statistical algorithm ADM-2 and a sensitivity threshold of 6.0. Significant copy-number changes were identified by at least three consecutive aberrant probes. Reference human genomic DNA was GRCh37/hg19.

### FISH analysis

Fluorescence in Situ Hybridization (FISH) analysis was performed on metaphase chromosomes to confirm array-CGH data. Commercial probes were selected for each patient and FISH was performed following the manufacturer’s protocol (Figure 2 legend). Slides were observed using a fluorescence microscope (Nikon, Eclipse 50i) and analyzed with Genikon software. Paternity was tested in *de novo* cases by microsatellite segregation.

### Consent

Written informed consent was obtained from the patient’s guardian/parent/next of kin for the publication of this report and any accompanying images.
